# A Cross-Sectional Study of the Knowledge, Practice, and Attitude Towards Herpes Zoster Vaccination Among the General Population in the Western Region of Saudi Arabia

**DOI:** 10.7759/cureus.33508

**Published:** 2023-01-08

**Authors:** Omar S Alhothali, Ammar S Alhothali, Azzam A Hanif, Mohammed F Bondagji, Hazem M Aljabri, Reda Goweda

**Affiliations:** 1 Faculty of Medicine, Umm Al-Qura University, Makkah, SAU; 2 Faculty of Family Medicine, Suez Canal University, Ismailia, EGY; 3 Faculty of Community Medicine, Umm Al-Qura University, Makkah, SAU

**Keywords:** vaccination campaign, herpes zoster virus, herpes zoster vaccine, vaccine, saudi arabia, attitude, knowledge, herpes zoster

## Abstract

Introduction

Herpes zoster (HZ) is a viral infection that occurs due to the reactivation of the varicella-zoster virus. The vaccination against herpes zoster to prevent its complications has been approved for individuals 50 years of age and older. This study aims to evaluate the knowledge, attitudes, and habits of at-risk populations about the varicella-zoster virus and its vaccination.

Methodology

A quantitative, observational, cross-sectional study was conducted among 500 adults over 50 years of age. Participants were selected by non-probability, convenience sampling from public places. RStudio (R version 4.1.1) was used to analyze the data.

Result

Eighty-three percent (n = 416) of participants had heard of herpes zoster (HZ). Seventy-four percent of respondents (n = 368) did not recognize the link between varicella and herpes zoster. Multiple linear regression showed that individuals who had varicella and heard about herpes zoster were the only positive predictors of herpes zoster knowledge. Out of all the respondents, 55.8% (n = 279) had heard of the herpes zoster vaccine, but 94.6% (n = 473) had not taken it. Among the respondents, 28.1% (n = 118) were unwilling to take optional vaccines; 77.4% (n = 387) agreed to take the HZ vaccine if recommended by a healthcare professional.

Conclusion

The general Saudi population had a good understanding of HZ and its vaccine. Their attitudes toward the HZ vaccine were generally positive; however, poor practices were observed. We recommend that arranging national campaigns targeting at-risk populations can enhance awareness about herpes zoster and its vaccine, subsequently increasing the rate of HZ immunization.

## Introduction

Herpes zoster (HZ), which results from the reactivation of varicella-zoster virus (VZV), is a viral infection that typically presents as painful grouped herpetiform vesicles that are highly contagious and cause significant health concerns worldwide [1,2]. VZV primarily affects children, causing varicella, known as chickenpox [3]. After the primary infection, the virus migrates and remains latent in the sensory dorsal root ganglia; the patient will be asymptomatic [4,5]. Herpes zoster, also called shingles, may present from a few weeks to many decades after VZV infection [4,6]. Therefore, HZ affects individuals who have had chickenpox [5].

The incidence of HZ is higher in patients with diabetes mellitus, immunosuppression, or over 60 years of age [4,5]. Herpes zoster is characterized by a vesicular skin eruption with a dermatomal distribution that crusts for around ten days [1]. Additionally, it is accompanied by burning, tingling, or aching pain [1,7]. Regarding the incidence of HZ, it affects approximately one million patients annually in the United States. The risk of being affected by HZ increased among persons over 60 [8,9]. Post-herpetic neuralgia (PHN) is one of the commonest complications of HZ, which is defined as persistent neuropathic pain for at least three months at the affected site after the onset of the rash. In addition, other complications may occur, such as herpes zoster ophthalmicus and HZ encephalitis, which impair social interaction and compromise the quality of life [10,11].

Antiviral drugs such as Acyclovir are used to decrease the severity and length of the disease. Analgesic medications are prescribed for the pain, whereas calamine lotion and wet compresses are used to relieve the itchiness [12]. Preventive medicine is considered the most effective way to save the health of the elderly, and vaccination against the most prevalent infectious diseases is the best-recommended strategy [13]. Shringrix, a two-dose recombinant HZ vaccine, was established in 2017 to prevent HZ reactivation and PHN and decrease the disease's intensity in case of relapse [14]. Shingrix can be administered to the general community and immunodeficient patients, and the vaccine's effectiveness reaches 90% [15].

The Centers for Disease Control and Prevention (CDC) recommend immunocompetent individuals over 50 years to receive two doses of Shingrix separated by two to six months, whether or not they report a previous episode of herpes zoster or a last dose of Zostavax. Also, it is not mandatory to screen for previous varicella infection, either through laboratory serology or verbally [16]. Patients who are or will be immunocompromised or immunosuppressed should receive the two doses at 19 years of age and older. The second dose is typically given two to six months after the first dose [16]. However, for persons who are or will be immunodeficient or immunosuppressed and who would benefit from completing the series in a shorter period, the second dose can be administered one or two months after the first dose [16].

To our knowledge, no previous study has evaluated the knowledge of HZ and its vaccine in Saudi Arabia. We aimed to investigate older people's knowledge, attitudes, and practices (KAP) on the HZ virus and its vaccine in Saudi Arabia.

## Materials and methods

Research design and sampling method

A quantitative, observational, cross-sectional study was carried out to assess the knowledge, attitude, and practices (KAP) of the Saudi Arabian population regarding the HZ virus and its vaccine. A non-probability, convenience sample of individuals over 50 who speak Arabic for easier communication was included. Visitors to Saudi Arabia were excluded from the study. We used OpenEpi (version 3.0) for sample size calculation: a minimum sample size of 385 was required for the study, considering a 95% confidence interval (CI), an anticipated frequency of 50%, and design effects of one.

Data collection tools and process 

A closed-ended structured questionnaire was adapted from a study carried out in the UAE [5]. There were 32 closed-ended questions in total, separated into the following four categories: demographics (10 questions), knowledge of HZ and its vaccination (14 questions), and attitudes (eight questions). It consisted of true and false, multiple choice, and Likert scale questions. Five hundred people in total were interviewed using convenience sampling in public places (shopping malls, parks, and beaches) encompassing Jeddah, Makkah, and Taif in November 2022. Those who were included in the study were asked to sign a consent form, and they were then interviewed using the online questionnaire that had been developed. No participant-identifying information was gathered to ensure confidentiality.

Scoring 

A knowledge score for participants' knowledge about shingles was calculated based on participants' correct responses to five questions. Two questions included multiple responses (six correct answers), whereas three had one correct answer for each. Therefore, the shingles knowledge score relied on nine selections, ranging between zero and nine. For the shingles vaccine knowledge, a score was calculated based on five questions. Each correct answer was assigned a score of one. Thus, a vaccine knowledge score ranged between zero and five.

Statistical analysis

Statistical analysis was carried out using RStudio (R version 4.1.1). We used frequencies and percentages to present categorical data, and numerical data were presented as the median and interquartile range (IQR). A multiple-response analysis was used to analyze variables with multiple selections. Group-based differences in participants' awareness and knowledge about shingles and the shingles vaccine were assessed using a Pearson's chi-squared test or a Fisher's exact test whenever applicable. Predictors of knowledge were assessed by constructing a multivariate linear regression analysis using the significantly associated factors from the group-based association analysis. Beta coefficients and 95% confidence intervals (95% CI) were used to present the outcomes of the regression analysis. A p-value of 0.05 indicated statistical significance.

Ethical considerations

This study was approved by the Medical Research Ethics Committee at Umm Al-Qura University (approval number: HAPO-02-k-012-2022-11-1223). Consent to participate was taken from participants electronically.

## Results

Sociodemographic characteristics 

We received 501 responses on the online platform. However, 500 responses were analyzed because one participant disagreed with participating. More than two-thirds of the sample were women (68.4%), and more than three-quarters of participants were aged 50 to 60 years (75.2%), were Saudis (86.8%), and were married (75.8%). Additionally, 56.8% of the respondents had obtained a university degree or higher, almost one-third of the respondents (34.6%) were employed (in the government or private sector), and 33.0% were retired. Only 4.2% of participants were working in the healthcare sector. Of note, 44.4% of the sample had at least one chronic condition; the most common conditions were hypertension (58.7%) and diabetes (54.0%). Importantly, 69.8% of the respondents had chicken pox (Table 1).

**Table 1:** Socio-demographic characteristics of the participants (N=500)

Parameter	Category	N (%)
Gender	Male	158 (31.6%)
	Female	342 (68.4%)
Age	50 to 60 years	376 (75.2%)
	> 60 years	124 (24.8%)
Nationality	Saudi	434 (86.8%)
	Non-Saudi	66 (13.2%)
Marital status	Single	28 (5.6%)
	Married	379 (75.8%)
	Divorced/Widow	93 (18.6%)
Educational level	Can read and write	47 (9.4%)
	Secondary school or less	169 (33.8%)
	University or higher	284 (56.8%)
Occupation	None	162 (32.4%)
	Employed	173 (34.6%)
	Retired	165 (33.0%)
Work in the healthcare sector	Yes	21 (4.2%)
Have a chronic condition	Yes	222 (44.4%)
Type of chronic condition(s)	Diabetes	115 (54.0%)
	Hypertension	125 (58.7%)
	Rheumatic disease	6 (2.8%)
	Asthma	8 (3.8%)
	Thyroid disease	14 (6.6%)
	Hypercholesterolemia	3 (1.4%)
	Cancer	2 (0.9%)
	Kidney disease	5 (2.3%)
	Hypercholesterolemia	4 (1.9%)
Have had chicken pox	Yes	349 (69.8%)
Have heard about shingles	Yes	416 (83.2%)

Awareness and knowledge regarding shingles

**Figure 1:**
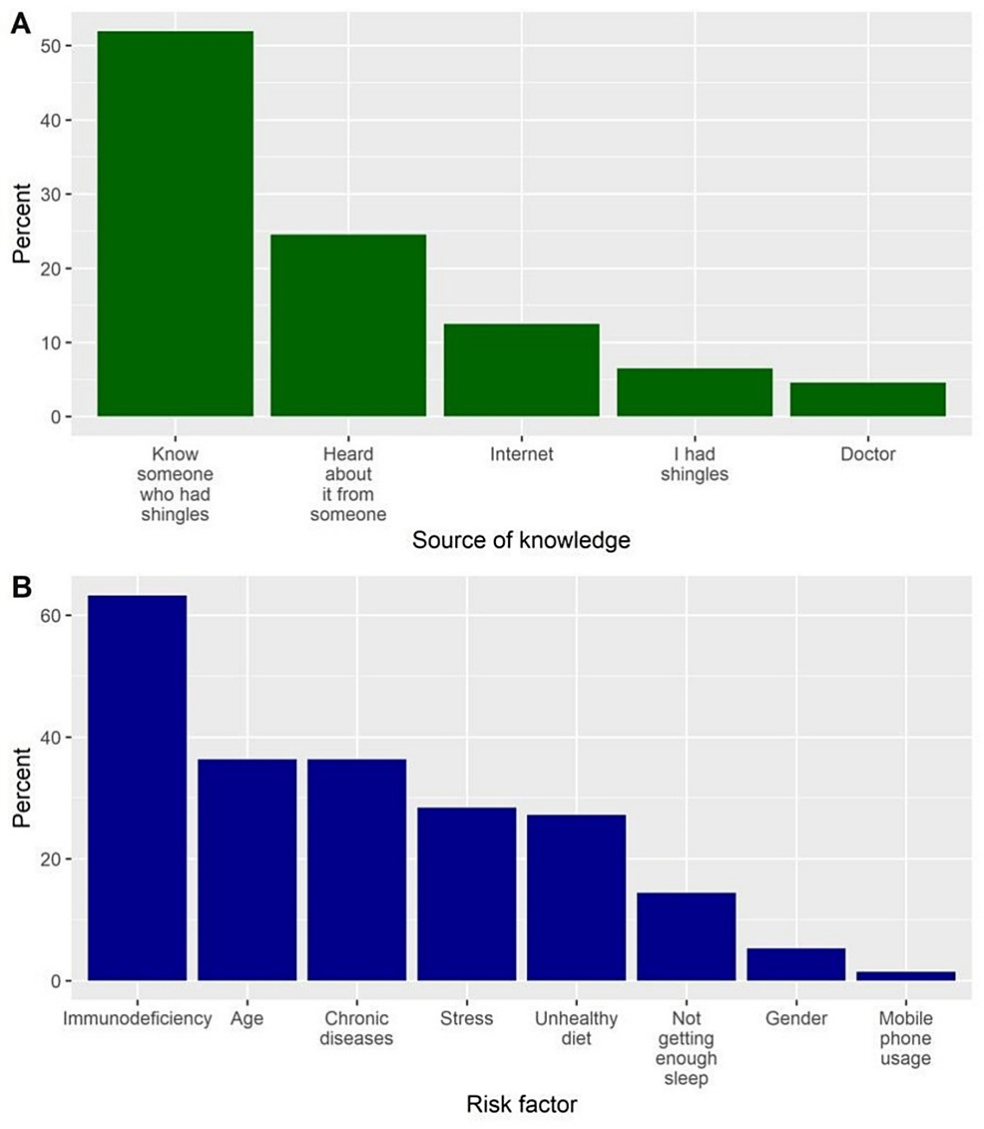
(A) The percentages of participants’ responses regarding the sources of knowledge about shingles; (B) The relevant risk factors

In general, 416 participants indicated that they had ever heard (were aware) about shingles, representing 83.2% (95% CI, 79.6 to 86.3). Focusing on aware participants, the most common sources of awareness were knowing someone who had shingles (51.9%) and having heard about shingles from someone (24.5%) (Figure 1A). Additionally, the most identifiable risk factors were immunodeficiency (63.2%), age (36.3%), and chronic diseases (36.3%) (Figure 1B).

Considering the factors that were associated with the awareness of the whole sample, the results showed that awareness about shingles was significantly higher among women (86.0% vs. 77.2% among men, p = 0.015), Saudis (86.6% vs. 60.6% among non-Saudis, p < 0.001) and those who had a history of chickenpox (85.7% vs. 77.5% among those who had not had chickenpox, p = 0.025). Additionally, participants' awareness increased consistently with higher educational levels (68.1% among those who could read and write, 75.1% among those who had a secondary school education or less, and 90.5% among those with a university degree or higher, p < 0.001). Furthermore, our research showed that participants' awareness increased with being employed (86.7%) or retired (88.5%) vs. not being employed (74.1%, p < 0.001). This is shown in Table 2.

**Table 2:** Factors associated with participants' awareness and knowledge regarding shingles and the shingles vaccine

Parameter	Category	Awareness about shingles		Knowledge score (of the disease)		Awareness about the shingles vaccine		Knowledge score (of the vaccine)	
		Yes, N = 416	p-value	Median (IQR)	p-value	Yes, N = 279	p-value	Median (IQR)	p-value
Gender	Male	122 (77.2%)	0.015	4.00 (3.00, 5.00)	0.250	94 (59.5%)	0.258	1.00 (0.00, 2.00)	0.988
	Female	294 (86.0%)		4.00 (3.00, 5.00)		185 (54.1%)		1.00 (0.00, 2.00)	
Age	50 to 60 years	315 (83.8%)	0.548	4.00 (3.00, 5.00)	0.165	212 (56.4%)	0.648	1.00 (0.00, 2.00)	0.245
	> 60 years	101 (81.5%)		4.00 (2.00, 5.00)		67 (54.0%)		1.00 (0.00, 2.00)	
Nationality	Saudi	376 (86.6%)	<0.001	4.00 (3.00, 5.00)	0.563	253 (58.3%)	0.004	1.00 (0.00, 2.00)	0.337
	Non-Saudi	40 (60.6%)		4.00 (3.00, 5.00)		26 (39.4%)		1.00 (0.00, 2.00)	
Marital status	Single	23 (82.1%)	0.668	3.50 (3.00, 5.00)	0.907	17 (60.7%)	0.367	1.00 (0.00, 2.00)	0.713
	Married	318 (83.9%)		4.00 (3.00, 5.00)		216 (57.0%)		1.00 (0.00, 2.00)	
	Divorced/Widow	75 (80.6%)		4.00 (3.00, 5.00)		46 (49.5%)		1.00 (0.00, 2.00)	
Educational level	Read and write	32 (68.1%)	<0.001	3.00 (2.00, 5.00)	0.012	15 (31.9%)	<0.001	1.00 (0.00, 2.00)	0.701
	Secondary school or less	127 (75.1%)		4.00 (2.00, 5.00)		75 (44.4%)		1.00 (0.00, 2.00)	
	University or higher	257 (90.5%)		4.00 (3.00, 5.00)		189 (66.5%)		1.00 (0.00, 2.00)	
Occupation	None	120 (74.1%)	<0.001	4.00 (2.00, 5.00)	0.057	69 (42.6%)	<0.001	1.00 (0.00, 2.00)	0.706
	Employed	150 (86.7%)		4.00 (3.00, 5.00)		112 (64.7%)		1.00 (0.00, 2.00)	
	Retired	146 (88.5%)		3.00 (2.00, 5.00)		98 (59.4%)		1.00 (0.00, 2.00)	
Work in the healthcare sector	No	398 (83.1%)	>0.999	4.00 (3.00, 5.00)	0.107	264 (55.1%)	0.141	1.00 (0.00, 2.00)	0.796
	Yes	18 (85.7%)		4.00 (3.00, 6.00)		15 (71.4%)		1.00 (1.00, 2.00)	
Have a chronic condition	No	230 (82.7%)	0.755	4.00 (3.00, 5.00)	0.830	155 (55.8%)	0.982	1.00 (0.00, 2.00)	0.095
	Yes	186 (83.8%)		4.00 (3.00, 5.00)		124 (55.9%)		1.00 (0.00, 2.00)	
Diabetes	No	325 (84.4%)	0.183	4.00 (3.00, 5.00)	0.226	216 (56.1%)	0.802	1.00 (0.00, 2.00)	0.094
	Yes	91 (79.1%)		4.00 (2.50, 5.00)		63 (54.8%)		1.00 (0.00, 2.00)	
Hypertension	No	311 (82.9%)	0.782	4.00 (3.00, 5.00)	0.814	210 (56.0%)	0.876	1.00 (0.00, 2.00)	0.434
	Yes	105 (84.0%)		4.00 (3.00, 5.00)		69 (55.2%)		1.00 (0.00, 2.00)	
Rheumatic disease	No	411 (83.2%)	>0.999	4.00 (3.00, 5.00)	0.062	275 (55.7%)	0.698	1.00 (0.00, 2.00)	0.930
	Yes	5 (83.3%)		5.00 (4.25, 5.75)		4 (66.7%)		1.00 (0.25, 1.75)	
Asthma	No	409 (83.1%)	>0.999	4.00 (3.00, 5.00)	0.400	273 (55.5%)	0.476	1.00 (0.00, 2.00)	0.808
	Yes	7 (87.5%)		3.00 (2.75, 4.00)		6 (75.0%)		1.00 (0.00, 3.00)	
Thyroid disease	No	402 (82.7%)	0.142	4.00 (3.00, 5.00)	0.391	271 (55.8%)	0.918	1.00 (0.00, 2.00)	0.656
	Yes	14 (100.0%)		4.00 (3.00, 5.75)		8 (57.1%)		1.00 (0.25, 2.00)	
Hypercholesterolemia	No	414 (83.3%)	0.425	4.00 (3.00, 5.00)	0.273	278 (55.9%)	0.586	1.00 (0.00, 2.00)	0.569
	Yes	2 (66.7%)		3.00 (2.00, 3.50)		1 (33.3%)		0.00 (0.00, 1.50)	
Cancer	No	414 (83.1%)	>0.999	4.00 (3.00, 5.00)	0.891	277 (55.6%)	0.506	1.00 (0.00, 2.00)	0.925
	Yes	2 (100.0%)		4.00 (3.00, 5.00)		2 (100.0%)		1.00 (1.00, 1.00)	
Kidney disease	No	413 (83.4%)	0.198	4.00 (3.00, 5.00)	0.765	278 (56.2%)	0.175	1.00 (0.00, 2.00)	0.526
	Yes	3 (60.0%)		4.00 (3.00, 4.00)		1 (20.0%)		2.00 (0.00, 3.00)	
Hypercholesterolemia	No	412 (83.1%)	>0.999	4.00 (3.00, 5.00)	0.510	276 (55.6%)	0.634	1.00 (0.00, 2.00)	0.955
	Yes	4 (100.0%)		3.00 (2.50, 3.75)		3 (75.0%)		1.00 (0.75, 1.50)	
Have had chicken pox	No	117 (77.5%)	0.025	3.00 (2.00, 4.00)	<0.001	84 (55.6%)	0.96	1.00 (0.00, 2.00)	0.859
	Yes	299 (85.7%)		4.00 (3.00, 5.00)		195 (55.9%)		1.00 (0.00, 2.00)	
Have heard about shingles	No	NA	NA	3.00 (1.00, 4.00)	<0.001	17 (20.2%)	<0.001	1.00 (0.00, 2.00)	0.318
	Yes	NA		4.00 (3.00, 5.00)		262 (63.0%)		1.00 (0.00, 2.00)	

The median (IQR) score of knowledge about shingles was 4.0 (3.0 to 5.0), with a minimum of 0.0 and a maximum of 8.0. The score differed significantly based on the education level (p=0.012), having a history of chickenpox (p<0.001), and having ever heard about shingles (p<0.001). On the multivariate regression analysis, we showed that higher knowledge scores of shingles were independently predicted by having heard about chickenpox (beta = 0.78, 95% CI, 0.47 to 1.09, p < 0.001) and having ever heard about shingles (beta = 0.95, 95% CI, 0.55 to 1.34, p < 0.001). This is captured in Table 3.

**Table 3:** Predictors of high knowledge regarding shingles

Parameter	Category	Beta	95% CI	p-value
Educational level	Can read and write	—	—	
	Secondary school or less	0.09	-0.43, 0.62	0.724
	University or higher	0.37	-0.14, 0.88	0.155
Have had chicken pox	No	—	—	
	Yes	0.78	0.47, 1.09	<0.001
Have heard about shingles	No	—	—	
	Yes	0.95	0.55, 1.34	<0.001

Awareness and knowledge regarding the shingles vaccine

A total of 279 participants were aware of the shingles vaccine (55.8%, 95% CI, 51.3-61.2). The proportions of participants who were aware of the shingles vaccine were significantly higher among Saudis (58.3% vs. 39.4% among non-Saudis, p = 0.004), those with a university education or higher (66.5% vs. 44.4% among those with secondary education or less, and 31.9% among those who could read and write, p< 0.001), and employed respondents (64.7% vs. 59.4% among retired and 42.6% among unemployed respondents, p < 0.001, Table 2). Regarding the knowledge score of the shingles vaccine, the score had a median (IQR) value of 1.0 (0.0 to 2.0) and minimum and maximum values of 0.0 and 0.5, respectively. We found no significant differences in knowledge regarding sociodemographic characteristics (Table 2). Therefore, we did not conduct a regression analysis for the knowledge scores about the shingles vaccine.

Participants' attitudes and practice toward shingles

**Figure 2:**
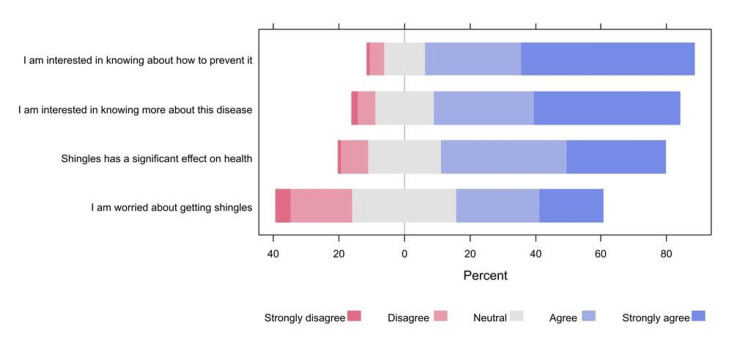
The percentages of participants’ responses regarding their attitudes and practices toward shingles

The majority of participants agreed or strongly agreed that they were interested in knowing about how to prevent shingles (82.2%) and about the disease (75.0%). Conversely, almost one-quarter of participants (23.4%) disagreed or strongly disagreed that they were worried about getting shingles (Figure 2).

Participants' attitudes and practices toward the shingles vaccine

Among the respondents, only 5.4% had received the shingles vaccine. However, most (77.4%) agreed or strongly agreed that they would get the vaccine if the doctor recommended it. Approximately one-third of the participants (29.4%) were worried about the cost of the vaccine. The most frequently reported barriers to get vaccinated are the lack of awareness about the vaccine (46.0%) and concerns about the possible side effects (20.2%) (Table 4).

**Table 4:** Participants’ attitudes and practices toward the shingles vaccine

Parameter	Category	N (%)
Ever had the shingles vaccine	No	473 (94.6%)
	Yes	27 (5.4%)
I would get the shingles vaccine if the doctor recommended it.	Strongly disagree	7 (1.4%)
	Disagree	14 (2.8%)
	Neutral	92 (18.4%)
	Agree	135 (27.0%)
	Strongly agree	252 (50.4%)
I worry about the cost of the vaccine.	Strongly disagree	79 (15.8%)
	Disagree	129 (25.8%)
	Neutral	145 (29.0%)
	Agree	91 (18.2%)
	Strongly agree	56 (11.2%)
What would prevent you from getting the shingles vaccine?	I am not at risk because I am healthy	93 (18.6%)
	I do not believe in vaccines	22 (4.4%)
	I would rather get medicine when I get sick	45 (9.0%)
	I am concerned about the side effects of the vaccine	101 (20.2%)
	I did not know that the vaccine existed	230 (46.0%)
	I believe it is a waste of money	9 (1.8%)

## Discussion

The topic of HZ has not yet been extensively researched in the Middle East and North Africa region. While several international studies have examined the impact of the HZ vaccine on the at-risk population and the reduction in economic burden resulting from vaccine administration, limited research has examined the population's willingness to take the vaccine and the barriers to vaccination.

There was overall good knowledge among the Saudi Arabian population regarding HZ. Over 80% of people in our sample were aware of HZ, while only about 50% knew about the HZ vaccine. This outcome is in line with research done in South Korea, where nearly half of the participants knew about the HZ vaccine and more than 80% of them knew about HZ. However, our results differ from a study done in the UAE population, where just 15% of people knew about the HZ vaccine, while just over 60% of people knew about HZ [5]. Additionally, a US study's findings show that participants' motivation to receive the vaccine was significantly influenced by their increased understanding of HZ and its vaccine [17].

Our results show that only less than 20% could answer most questions correctly. In addition, about 74% of the participants could not recognize the link between chickenpox and HZ. This may be due to the fact that more than 80% of participants learned about HZ from friends, family, or the internet, which are not the most reliable sources of information. Furthermore, postgraduates were better informed than others regarding HZ, consistent with a study from Hong Kong and the United Arab Emirates that found higher levels of education were associated with better HZ skills [4,5]. This illustrates the importance of education and providing the public with primary sources of information to minimize the spread of distorted facts and untruths.

The vaccination rates for HZ are extremely low in many different geographical areas, as demonstrated by many studies. In Saudi Arabia, only around 5% received the HZ vaccine. Only about 3% of people in the UAE and Hong Kong, and about 8% in the US, have received the HZ vaccine [4,5,17]. In our research, most participants were unaware that the vaccine was recommended for those over 50. This, combined with the fact that over 75% of participants showed positive attitudes and were willing to get the vaccine if a health care provider (HCP) recommended it, offers a possible strategy for encouraging individuals who are most in need of the vaccine to receive it.

While a South Korean study found that the cost of the HZ vaccine was a major barrier to vaccination [4], we found that cost was not a barrier in Saudi Arabia. We also found that about 60% of the participants didn't know about the existence of the HZ vaccine. This could be secondary to decreased interaction with HCPs, leading to a lack of knowledge about HZ and its vaccination.

Limitations 

This study has a few limitations. First, recall bias may have influenced the results. Participants were asked to self-report their previous history of chickenpox infection. Second, the study included only one region in Saudi Arabia, which may limit the generalizability of the results.

## Conclusions

Herpes zoster is a viral infection that occurs due to the reactivation of the varicella-zoster virus. The Saudi Ministry of Health recently recommended primary health centers administer HZV to adults aged over 50 years. The Saudi Arabian population's knowledge of HZ and its vaccine was good, and observed attitudes were positive. We would like to highlight the importance of these results to doctors, who often forget to discuss this subject with patients who are willing to be vaccinated. Nationwide campaigns highlighting the disease, its complications, and the importance of HZ vaccination could inspire the target population to improve their vaccination readiness.
